# MRI-based multiregional radiomics for predicting lymph nodes status and prognosis in patients with resectable rectal cancer

**DOI:** 10.3389/fonc.2022.1087882

**Published:** 2023-01-04

**Authors:** Hang Li, Xiao-li Chen, Huan Liu, Tao Lu, Zhen-lin Li

**Affiliations:** ^1^ Department of Radiology, Sichuan Academy of Medical Sciences and Sichuan Provincial People’s Hospital, Chengdu, Sichuan, China; ^2^ Department of Radiology, Affiliated Cancer Hospital of Medical School, University of Electronic Science and Technology of China, Sichuan Cancer Hospital, Chengdu, China; ^3^ GE Healthcare, Shanghai, China; ^4^ Department of Radiology, West China Hospital, Sichuan University, Chengdu, China

**Keywords:** lymph node, rectal neoplasms, magnetic resonance imaging, radiomics, prognosis

## Abstract

**Purpose:**

To establish and evaluate multiregional T2-weighted imaging (T2WI)-based clinical-radiomics model for predicting lymph node metastasis (LNM) and prognosis in patients with resectable rectal cancer.

**Methods:**

A total of 346 patients with pathologically confirmed rectal cancer from two hospitals between January 2019 and December 2021 were prospectively enrolled. Intra- and peritumoral features were extracted separately, and least absolute shrinkage and selection operator regression was applied for feature selection. Radiomics signatures were built using the selected features from different regions. The clinical-radiomic nomogram was developed by combining the intratumoral and peritumoral radiomics signatures score (radscore) and the most predictive clinical parameters. The diagnostic performances of the nomogram and clinical model were evaluated using the area under the receiver operating characteristic curve (AUC). The prognostic model for 3-year recurrence-free survival (RFS) was constructed using univariate and multivariate Cox analysis.

**Results:**

The intratumoral radscore (radscore 1) included four features, the peritumoral radscore (radscore 2) included five features, and the combined intratumoral and peritumoural radscore (radscore 3) included ten features. The AUCs for radscore 3 were higher than that of radscore 1 in training cohort (0.77 vs. 0.71, *P*=0.182) and internal validation cohort (0.76 vs. 0.64, *P*=0.041). The AUCs for radscore 3 were higher than that of radscore 2 in training cohort (0.77 vs. 0.74, *P*=0.215) and internal validation cohort (0.76 vs. 0.68, *P*=0.083). A clinical-radiomic nomogram showed a higher AUC compared with the clinical model in training cohort (0.84 vs. 0.67*, P*<0.001) and internal validation cohort (0.78 vs. 0.64, *P*=0.038) but not in external validation (0.72 vs. 0.76, *P*=0.164). Multivariate Cox analysis showed MRI-reported extramural vascular invasion (EMVI) (HR=1.099, 95%CI: 0.462-2.616; *P*=0.031) and clinical-radiomic nomogram-based LNM (HR=2.232, 95%CI:1.238-7.439; *P*=0.017) were independent risk factors for assessing 3-year RFS. Combined clinical-radiomic nomogram based LNM and MRI-reported EMVI showed good performance in training cohort (AUC=0.748), internal validation cohort (AUC=0.706) and external validation (AUC=0.688) for predicting 3-year RFS.

**Conclusion:**

A clinical-radiomics nomogram exhibits good performance for predicting preoperative LNM. Combined clinical-radiomic nomogram based LNM and MRI-reported EMVI showed clinical potential for assessing 3-year RFS.

## Introduction

Rectal cancer ranks eighth among all cancers worldwide (1). Lymph node metastasis (LNM) has been confirmed to be a poor prognostic factor in rectal cancer (2, 3). Preoperative prediction of LNM can provide useful information for determining the need for adjuvant therapy or surgical resection. Therefore, an accurate prediction of LNM plays an important role in clinical decision-making and improved prognosis (2, 4). Traditional imaging methods mainly focus on the size, shape and edge of lymph nodes to determine the lymph node status. However, these morphological features alone are not sufficient to reliably identify LNM in rectal cancer because reactive or inflammatory lymph nodes can be enlarged, normal-sized, or even small and account for a significant proportion of malignancy (5–7). An alternative technical approach is needed to complement the routine imaging tools used in the assessment of LNM.

Radiomics is a noninvasive method that allows the extraction of quantitative features from medical images (8). Several studies reported that CT- or MRI-based radiomics features could predict LNM in other malignant tumors (9–11). For rectal cancer, some studies reported that CT or MRI radiomics signature-based nomograms of the primary tumor have attained the ability to discriminate colorectal cancer patients with or without LNM (12–14). However, these previous reports only measured intratumoral regions, and the peritumoral region, which may contain valuable information about the tumor, was excluded. Tumor heterogeneity is not only solely limited to cancer cells but also relates to nonmalignant and infiltrating cells surrounding the tumor, commonly referred to as the microenvironment. It is the interaction between tumor cells and the surrounding microenvironment that influences tumor evolution and progression (15). Several studies have shown that radiomics based on peritumoural regions improves the diagnostic performance for identifying LNM in other cancers (16–18). Therefore, we can presume that radiomics derived from intratumoral and peritumoral regions could also predict LNM in rectal cancer. Furthermore, previous studies had small sample sizes and lacked complete external validation cohorts. To the best of our knowledge, there has been no study investigating associations between preoperative MRI-radiomics signatures on LNM and 3-year recurrence-free survival (RFS). A recently study reported that the advantage of radiomics offering better disease characterization might allow better performance of radiomics models based on T2WI alone, that is, without combining with diffusion weighted imaging (DWI) (19). Therefore, the purpose of this study was to develop and validate a T2WI-based clinical-radiomics model from intratumoral and peritumoral tissues for the preoperative prediction of LNM and prognosis in patients with resectable rectal cancer using a multicenter database.

## Materials and methods

### Study population

**Figure 1:**
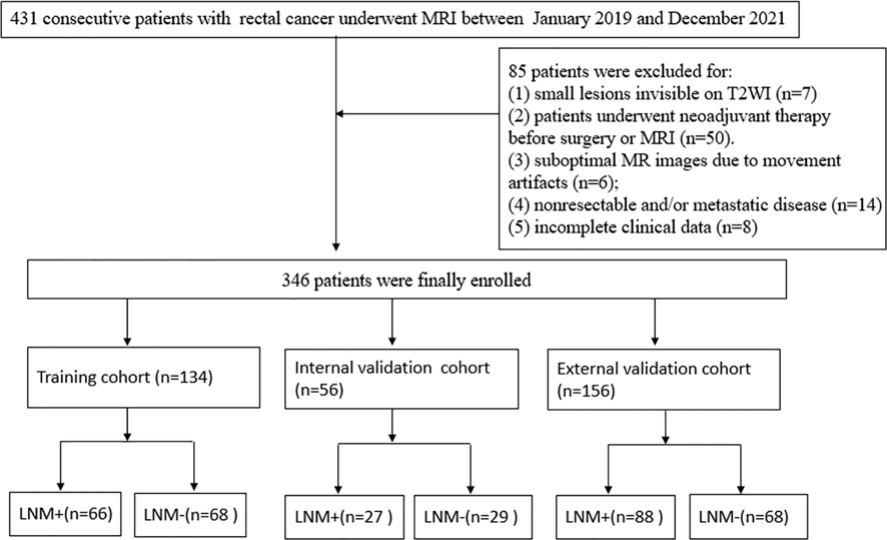
Flowchart of patient selection.

This prospective study was approved by the institutional review board in our institution, and the requirement for informed patient consent was obtained. From January 2019 to December 2021, we prospectively recruited 431 patients with rectal cancer from two hospitals who underwent radical surgery. We included the following patients: (1) patients who underwent MRI examination two weeks before surgery; (2) rectal adenocarcinoma diagnosis based on pathology of surgical specimens; and (3) 12 or more regional lymph nodes in the surgical specimen that needed to be examined. We excluded the following lesions: (1) small lesions invisible on T2-weighted images (T2WI) (n=7); (2) patients underwent neoadjuvant therapy before surgery or MRI (n=50). (3) suboptimal MR images due to movement artifacts or poor resolution (n=6); (4) nonresectable and/or metastatic disease (n=14); and (5) incomplete clinical data, such as lack of presurgical carcinoembryonic antigen [CEA] data (n=8). These patients were divided into three groups, namely, the training cohort (n=134) from hospital 1, the internal validation cohort (n=56) from hospital 1 at a ratio of 7:3 based on the scanning date, and an external validation cohort (n=156) from hospital 2. A flowchart of the study population is shown in **Figure 1**.

### MRI acquisition

A 1.5-T MR scanner (MAGNETOM Aera, Siemens Healthineers) was used at hospital 1, whereas a 3.0-T MR scanner (MAGNETOM Skyra, Siemens, Healthineers) was used at hospital 2. Before the MRI scan, 20 mg of scopolamine butylbromide (Buscopan, Boehringer Ingelheim) was intramuscularly injected to reduce bowel motion. Rectal distention was not performed before MR examinations. For hospital 1, the axial T2WI (perpendicular to the long axis of the rectum) without fat saturation was performed with the following protocol: TR/TE, 4600/75; field of view (FOV), 220 mm^2^; matrix size, 256 × 512; and 3-mm thickness without an interslice gap. For hospital 2, the following protocol was employed: TR/TE, 4960/89; FOV, 200 mm^2^; matrix size, 320 × 320; and 3-mm thickness without an intersection gap.

### MRI evaluation

Two radiologists (the first author and second author with 5 years and 12 years of experience in reporting rectal cancer MRI, respectively) blinded to the histopathology results reviewed the MR images in consensus. The tumor length and tumor thickness were measured on the sagittal and oblique axis T2WIs, respectively. extramural vascular invasion (EMVI) positivity on MRI was defined as follows: (1) tumor signal intensity in a vascular structure, (2) dilated vessels, and (3) tumoral extension through the vessel wall invading the vessel border. Qualitative criteria of MRI-reported lymph node metastasis were based on the 2016 European Society of Gastrointestinal and Abdominal Radiology consensus meeting (7). Disagreements between two radiologists in the assessment of these features were resolved through discussion.

### Tumor segmentation and feature extraction

**Figure 2:**
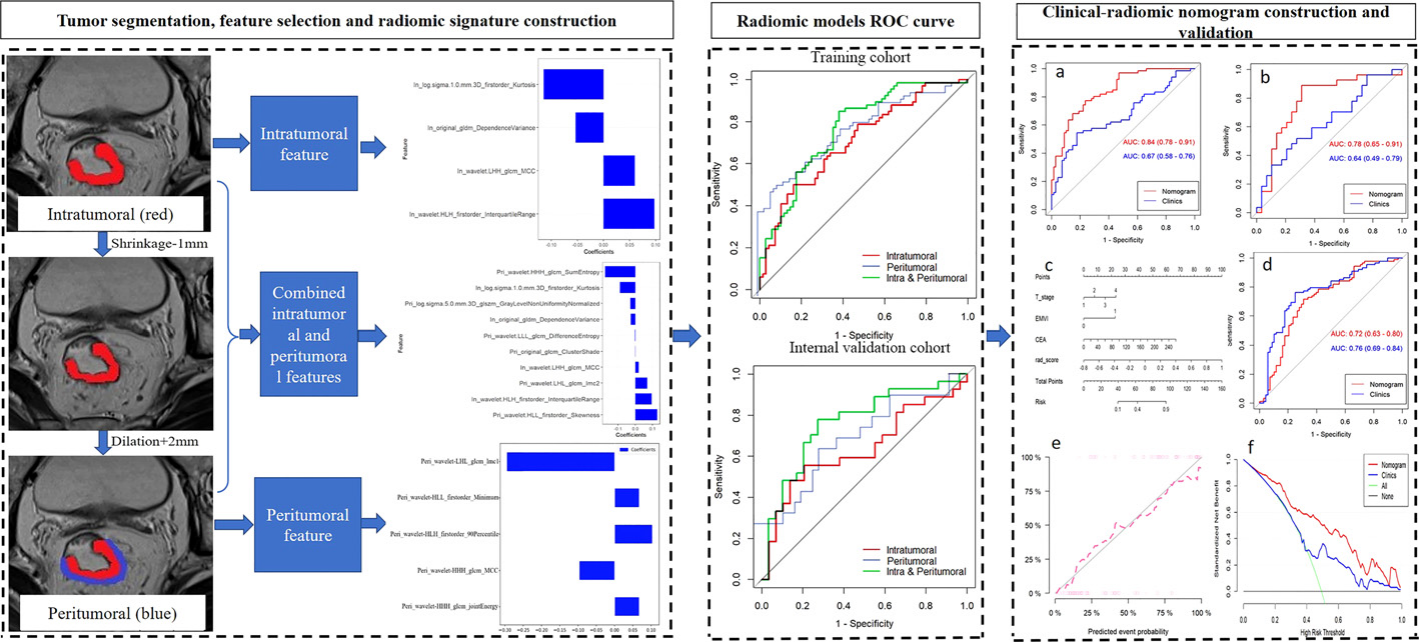
The workflow of a typical radiomics process in our study included tumor segmentation, feature selection, and model construction and evaluation.

A flowchart of the radiomics process is shown in **Figure 2**. One radiologist (the first author) segmented the volumes of interest of tumors on T2WI images with the AK software (Artificial Intelligence Kit, version 3.3.0, GE Healthcare) blinded to the histopathology results and a senior author (the last author) with 20 years’ experience scrutinized them. To acquire information at the invasive margin, peritumoral regions were obtained with automated dilation of the tumor boundaries by 2 mm on the outside and shrinkage of the tumor boundaries by 1 mm on the inside, resulting in a ring with a thickness of 3 mm (20). We carefully excluded obvious vessels, peritumoral organs, and air cavities. Intraclass correlation coefficients (ICCs) were calculated to assess the interobserver correlation coefficient reproducibility of the radiomic feature extraction. The reproducibility of radiomic features between two observers (the first author and second author) was evaluated with ICC based on the first 30 patients’ data. The subsequent feature extraction was performed by a radiomic module (backed by Pyradiomics) embedded in the open-source software package 3D Slicer (version 4.9, 107 http://www.slicer.org). Gray level of T2WI was quantized to 25 gray levels. Seven radiomic features categories included 14 first-order statistical features, 18 shape-based features, 22 gray level co-occurrence matrix, 16 gray level size zone matrix, 16 gray level run length matrix, 14 gray level dependence matrix, and 5 neighboring gray tone difference matrix. Moreover, two image filters, wavelet and Laplacian of Gaussian were applied to original images, respectively. Before the feature extraction, z score normalization of the MRI signal intensities for T2WI. Consequently, 1409 features were obtained for each of intratumoral region and peritumoral region. The time required for a senior radiologist to take segmentation was controlled to 300 seconds while for a junior radiologist to take segmentation was controlled to 600 seconds.

### Feature selection and model building

The values of the features with ICC >0.75 were included for subsequent analysis. ComBat harmonization was first used to remove batch effects that caused by the handling of samples by different centers or different scanner/protocol that can obscure individual variations (21). Feature selection and model building were performed using R software (version 2.15.3 www.r-114project.org).

The radiomics features were initially screened by maximum relevance and minimum redundancy, and then least absolute shrinkage selection operator regression was used to select the most useful predictive features from the training cohort. A radiomics signature score (radscore) was calculated for each patient as a linear combination of the selected features weighted by their respective coefficients. The predictive accuracy of the radscore was evaluated by the area under the curve (AUC) in the training and validation cohorts. The highest AUC value among the radscores was included in the subsequent analysis.

Wilcoxon test was first applied to all clinical risk factors and radscore, and then the factors with *P*<0.05 in univariate logistic regression was performed to choose the independent predictors. Multivariate logistic regression analysis was performed to construct the combined model. The nomogram and clinical model for predicting LNM were constructed using the selected predictors. The Hosmer–Lemeshow test was performed to assess the goodness-of-fit of the nomogram. Calibration curves were generated to evaluate the calibration of the nomogram. The AUC was calculated to assess the discrimination performances of the clinical model and the nomogram for predicting LNM. The clinical utility of the nomogram was evaluated by decision curve analysis (DCA).

### Outcome

Patients with rectal cancer at pathological T1-2N0M0 after surgery received “follow-up and watch” strategy, without giving any adjuvant treatment. For patients at pathological T3-4N0M0 or T1-4N1-2M0 after surgery, these patients received 5-fluorouracil-based adjuvant therapy. Relapse was assessed every 3–6 months based on clinical or radiological locoregional or distant progression after surgery. The primary endpoint was 3-year RFS.

### Statistical analysis

SPSS 23.0 (IBM) and R software were used for statistical analysis. The baseline characteristics of patients with rectal cancer were compared using Student’s t test, nonparametric test, chi-squared test, and Fisher’s exact test (where appropriate). The diagnostic performance was compared by ROC analysis, and the difference in AUCs between these models was compared using Delong’s test. The prognostic model for 3-year RFS was constructed using univariate and multivariate Cox analysis.

## Results

### Patient characteristics

A total of 346 patients (mean age 61.86 years, age range 26-88 years) were included in this study population. Among the 346 patients with rectal cancer, 134 patients were in the training cohort (66 pathologically reported LNM+ and 68 LNM-), 56 patients were in the internal validation cohort (27 pathologically reported LNM+ and 29 LNM-), and 156 patients were in the external validation cohort (88 pathologically reported LNM+ and 68 LNM-). Among these three cohorts, significant differences were found in MRI-reported EMVI (*P*= 0.031) and tumor length (*P*=0.030), as shown in **Table 1**.

**Table 1:** Baseline characteristics of the study population.

Characteristics	Training cohort(n=134)	Internal validation cohort (n=56)	External validation cohort (n=156)	*P* value
Sex				0.321
Male	93(69.4)	34(60.7)	97(62.2)	
Female	41(30.6)	22(39.3)	59(37.8)	
Age (mean± SD)	62.7 ± 10.6	62.1 **±** 12.5	61.1 ± 10.2	0.742
Location				0.180
upper	43(32.1)	14(25)	40(25.6)	
middle	57(42.5)	32(57.1)	82(52.5)	
lower	34(25.4)	10(17.9)	34(21.9)	
cT stage				0.052
T1	2(1.5)	2(3.6)	4(2.6)	
T2	15(11.2)	8(14.3)	30(19.2)	
T3	79(59)	21(37.5)	96(61.5)	
T4	38(28.3)	25(44.6)	26(16.7)	
MRI-reported LNM				0.122
Negative	71(53)69	21(37.5)	81(51.9)	
Positive	63(47)65	35(62.5)	75(48.1)	
MRI-reported EMVI				0.031
Negative	99 (73.9)	50 (89.3)	76(48.7)	
Positive	35 (26.1)	6 (10.7)	80(51.3)	
CA199(mean± SD)	40.7 ± 152.6	19 ± 53.7	30.7 ± 87.8	0.300
CEA (mean± SD)	10.8 ± 25.3	6 ± 14.4	11.1 ± 24	0.182
Tumor length (mean± SD)	45.6 ± 16.7	40.3 ± 12	41.6 ± 18.6	0.030
Tumor thickness (mean± SD)	14.2 ± 6.7	12.5 ± 4.5	30.2 ± 13.4	0.078

SD, standard deviation; MRF, mesorectal fascia; LNM, lymph node metastasis; EMVI, extramural vascular invasion; CEA, carcinoembryonic antigen; CA199, carbohydrate antigen 199.

### MR-reported LNM correlation with pathologic results

MR-reported LNM correlation with pathologic results is summarized in **Supplementary Table 1**. The correlation of MR-reported LNM with pathologic results were validated with Kappa of 0.248, with a sensitivity of 62.4% and specificity of 62.4%. Therefore, the correlation of MR-reported LNM with pathologic results indicated poor consistency because the Kappa value was less than 0.4.

### Radscore evaluation

**Figure 3:**
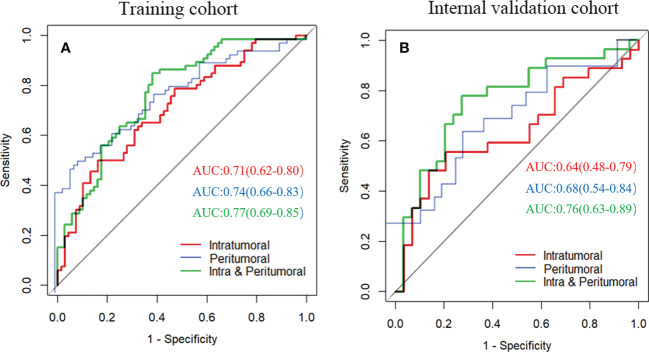
Receiver operating characteristic curves of three radiomics models for predicting lymph node metastasis in training cohort **(A)** and internal validation cohort **(B)**.

The intratumoral radscore (radscore 1) included four features. The peritumoral radscore (radscore 2) included five features. The combined intratumoral and peritumoural radscore (radscore 3) included ten features **(** **Supplementary Figure 1** **)**. The ROC curves of radscore 1, radscore 2 and radscore 3 were generated for predicting LNM in training and internal validation cohorts **(** **Figure 3** **)**. The radscore 3 achieved a higher AUC compared with radscore 1 in training cohort (0.770 vs. 0.710, *P*=0.182) and internal validation cohort (0.760 vs. 0.640, *P*=0.041). The AUCs for radscore 3 were higher than that of radscore 2 in training cohort (0.77 vs. 0.74, *P*=0.215) and internal validation cohort (0.76 vs. 0.68, *P*=0.083).

### Development and evaluation of the clinical-radiomic nomogram

**Figure 4:**
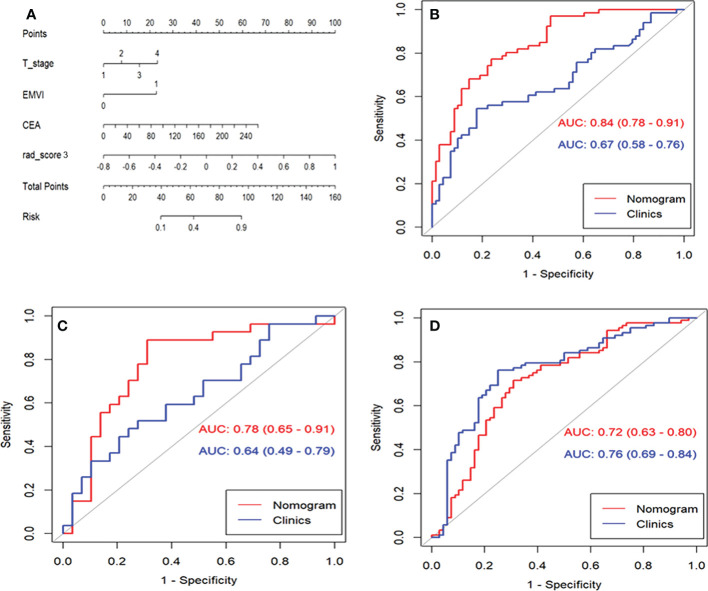
The performance and validation of the final selected model to predict lymph node metastasis (LNM). **(A)**, The predictive nomogram of LNM in training cohort. **(B)**, Receiver operating characteristic curves (ROC) of clinical model and nomogram to predict LNM with rectal cancer in training cohort. **(C)**, ROC of clinical model and nomogram to predict LNM with rectal cancer in internal validation cohort. **(D)** ROC of clinical model and nomogram to predict LNM with rectal cancer in external validation cohort.

**Figure 5:**
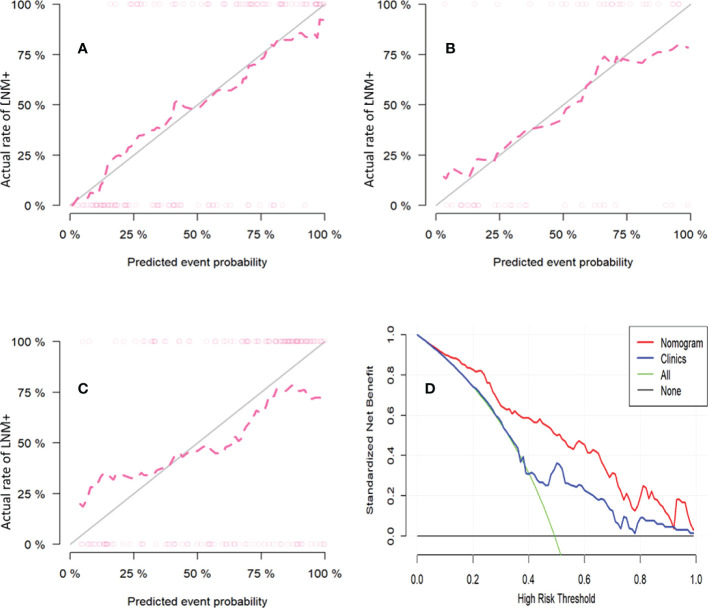
The calibration curves for the nomogram in training cohort **(A)**, internal validation cohort **(B)** and external validation cohort **(C)**. The diagonal gray line represents a perfect prediction by an ideal model. The pink dotted line represents the performance of the nomogram, of which a closer fit to the diagonal gray line represents a better prediction (Hosmer-Lemeshow test all p-values >0.05). Decision curve analysis of nomogram to investigate the clinical usefulness in predicting lymph node metastasis **(D)**. It indicates the nomogram model obtains more benefit than “treat all”, “treat none”, and the clinical model when the threshold probability is >10% in training cohort.

A clinical model was constructed using three factors, including cT stage, MRI-reported EMVI, and CEA. The clinical-radiomic combined model was constructed by adding radscore 3 to the clinical model [odds ratio (OR)=1.566 for radscore 3, 1.841 for cT, 8.340 for EMVI, and 1.020 for CEA], as summarized in **Table 2**. The nomogram was constructed for visualizing the combined model, as shown in **Figure 4**. The calibration curves and DCA results of the clinical-radiomics nomogram are shown in **Figure 5**. Good calibration in training cohort and validation cohort was identified using the Hosmer–Lemeshow test (all *P*>0.05).

**Table 2:** Univariate and multivariate logistic regression analysis for clinical characteristics and radiomic signature.

Parameters	Univariate analysis		*P* value	Multivariate analysis		*P* value
	OR	95 %CI		OR	95% CI	
Radscore 3	1.437	1.238-1.667	**<0.001**	1.556	1.307-1.852	**<0.001**
Gender	1.572	0.748-3.304	0.233			
Age	0.982	0.950-1.014	0.265			
Location	0.745	0.474-1.173	0.204			
cT-stage	1.861	1.074-3.224	**0.027**	1.841	0.914-3.706	**0.038**
MRI-reported LNM	1.609	0.813-3.185	0.172			
MRI-reported EMVI	4.261	1.807-10.047	**0.001**	8.340	2.898-24.001	**<0.001**
CA199	1.005	0.998-1.011	0.190			
CEA	1.040	1.003-1.078	**0.035**	1.020	0.981-1.062	**0.045**
Tumor-length	1.013	0.992-1.034	0.240			
Tumor thickness	0.985	0.936-1.037	0.576			

EMVI, extramural venous invasion; LNM, lymph node metastasis; CEA, carcinoembryonic antigen; CA199, carbohydrate antigen 199; OR, odds ratio; CI, confidence interval; radscore 3, combined intratumoral and peritumoral radiomic signature score. The bold for P-value means there is a significant difference.

The AUCs for the clinical model were 0.67 (95% CI: 0.58-0.76) and 0.64 (95% CI: 0.49-0.79) in training cohort and internal validation cohort, respectively **(** **Table 3** **)**. AUCs were improved by adding the clinical model to the radscore 3. The AUCs for the clinical-radiomic nomogram were higher than that of the clinical model in training cohort (0.84 vs. 0.67, *P*<0.001) and internal validation cohort (0.78 vs. 0.64, *P*=0.038). However, the nomogram failed to outperform the clinical model in external validation cohort (0.72 vs. 0.76, *P*=0.164).

**Table 3:** Diagnostic performance of the radiomics model, clinical model, and the clinical-radiomic nomogram.

Data set	Model	AUC (95%CI)	Sensitivity	Specificity
Training cohort	Radscore 1	0.71(0.62-0.80)	0.500	0.838
	Radscore 2	0.74 (0.66-0.83)	0.712	0.632
	Radscore 3	0.77 (0.69-0.85)	0.848	0.618
	Clinical model	0.67 (0.58-0.76)	0.545	0.823
	Nomogram	0.84 (0.78-0.91)	0.773	0.765
Internal validation	Radscore 1	0.64 (0.48-0.79)	0.592	0.586
	Radscore 2	0.68 (0.54-0.84)	0.700	0.619
	Radscore 3	0.76 (0.63-0.89)	0.889	0.414
	Clinical model	0.64 (0.49-0.79)	0.296	0.896
	Nomogram	0.78 (0.65-0.91)	0.719	0.833
External validation	Clinical model	0.76 (0.69-0.84)	0.761	0.720
	Nomogram	0.72 (0.63-0.80)	0.456	0.818

CI, confidence intervals; AUC, the area under the receiver operating characteristic curve; Radscore 1, intratumoral radiomic signature score; Radscore 2, peritumoral radiomic signature score; Radscore 3, combined intratumoral and peritumoral radiomic signature score.

### Subgroup analyses

**Figure 6:**
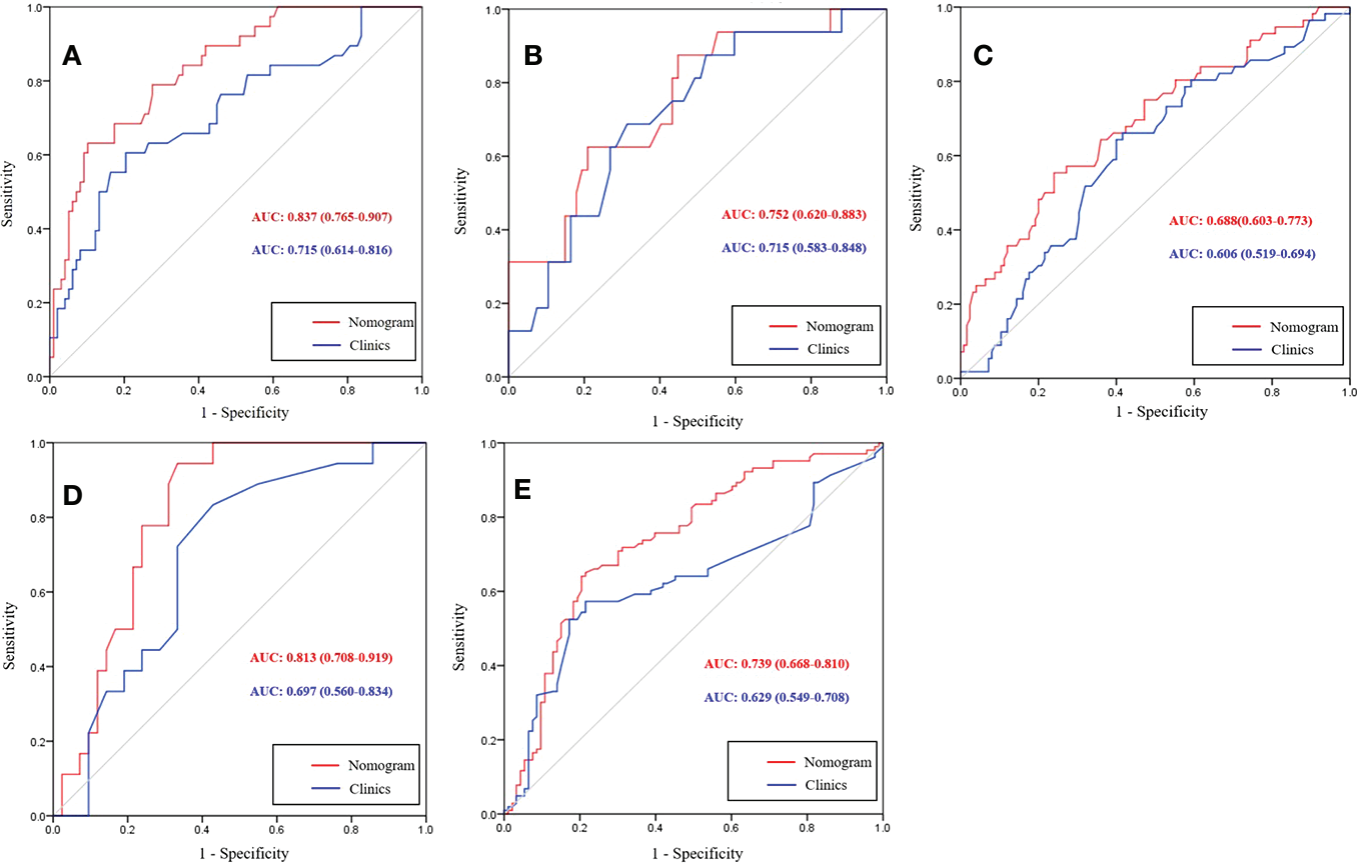
Receiver operating characteristic (ROC) curves of the nomogram and clinical model for differentiating extranodal extension (ENE) positive from lymph node metastasis (LNM) negative **(A)**, lateral lymph node positive from LNM negative **(B)**, and N2 stage from LNM negative **(C)**. At T1-T2 stage subgroup analysis, ROC curves of nomogram and clinical model for predicting LNM **(D)**. At T3 stage subgroup analysis, ROC curves of a clinical-radiomics nomogram and clinical model for predicting LNM **(E)**.

Subgroup analyses of the nomogram are shown in **Figure 6**. Extranodal extension (ENE), which is defined as the extension of tumor cells through the nodal capsule into the perinodal fatty tissue, is an adverse prognostic factor in rectal cancer (22–24). Pathological specimens of 93 patients with LNM were reviewed by a pathologist to determine the ENE status. In total, 38 patients were ENE positive. The nomogram had a higher AUC than the clinical model for identifying ENE (0.837 vs. 0.715, *P*=0.004). Lateral lymph node metastasis (LLNM) has a significantly higher risk of lateral pelvic recurrence compared to those who had negative LLNM. LLNM is considered as distant metastasis that is treated with neoadjuvant chemoradiotherapy (nCRT) followed by surgery. Forty patients underwent dissection. There were 16 patients with LLNM confirmed by pathological specimens. Although the nomogram had a higher AUC than the clinical model for identifying LLNM, the difference was not significant (0.752 vs. 0.715, *P*=0.538). Patients with N2 stage had worse prognosis than the patients with N0 and N1 stage. In total, 56 patients with N2 stage were confirmed by pathological specimen. The nomogram had a higher AUC than the clinical model for differentiating N2 stage from LNM-negative patients, but the difference was not significant (0.688 vs. 0.606, *P*=0.109). For patients at T1-T2 stage without LNM, these patients can receive surgery without giving preoperative nCRT. At T1-T2 stage patients, there were 18 patients with LNM and 42 patients without LNM. The nomogram had a higher AUC than the clinical model for identifying LNM (0.813 vs. 0.697, *P*=0.034). For patients at T3 stage without high risk factors such as LNM, these patients can receive surgery and then giving postoperative adjuvant therapy. At T3 stage patients, there were 103 patients with LNM and 93 patients without LNM. The nomogram had a higher AUC than that of the clinical model for identifying LNM (0.739 vs. 0.629, *P*=0.003).

### Survival analysis

**Figure 7:**
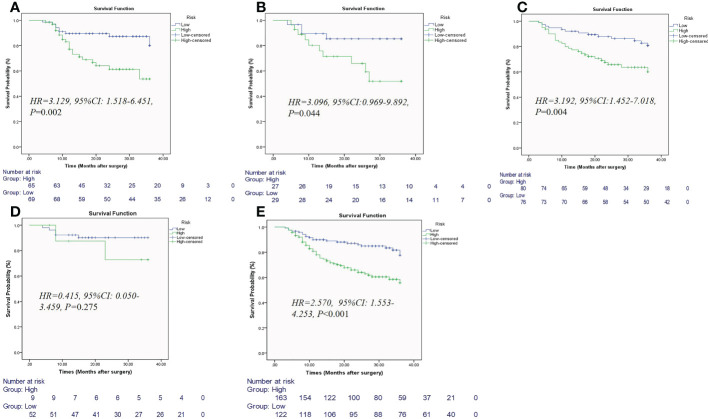
Kaplan-Meier estimates of the nomogram for predicting 3-year recurrence-free survival in patients with rectal cancer in training cohort **(A)**, internal validation cohort **(B)**, external validation cohort **(C)**, at T1-T2 subgroup **(D)**, and at T3-T4 subgroup **(E)**.

The median follow-up in the event-free population was 26 months (range, 5–36 months) in training cohort, 26 months (range, 6–36 months) in testing cohort, and 36 months (range, 5–36 months) in external validation cohort. The rate of recurrence in patients with LNM was higher than that of those without LNM (31.8% vs. 16.9%). Among the 134 patients in the training cohort, locoregional or distant relapse occurred in 30 patients (22.4%) after a median duration of 21 months (4–36 months). Among the 56 patients in validation cohort, locoregional or distant relapse occurred in 14 patients (25%) after a median duration of 22 months (4–36 months). Among the 156 patients in external validation cohort, locoregional or distant relapse occurred in 41 patients (26.2%) after a median duration of 31.5 months (3–36 months). Kaplan−Meier survival curves showed that the patients with low clinical-radiomic nomogram score had better 3-year RFS than those with high scores in training cohort, internal validation cohort, external validation cohort, and at T3-T4 stage (all *P*<0.05) (**Figure 7**). In the training cohort, univariate Cox analysis revealed age, MRI-reported EMVI and clinical-radiomic nomogram-based LNM were correlated with 3-year RFS (all *P*<0.05). Multivariate Cox analysis showed MRI-reported EMVI (HR=1.099, 95%CI: 0.462-2.616; *P*=0.031) and clinical-radiomic nomogram-based LNM (HR=2.232,95%CI:1.238-7.439; *P*=0.017) were independent risk factors for 3-year RFS (**Table 4**). The prognostic model for 3-year RFS prediction was constructed with MRI-reported EMVI and clinical-radiomic nomogram-based LNM and indicated good performance, with AUC of 0.748 in training cohort, 0.706 in internal validation and 0.688 in external validation cohort.

**Table 4:** Univariate and multivariate Cox analysis of 3-year recurrence-free survival based on training cohort.

Variate	Univariate		Multivariate	
	*P*	HR (95%CI)	*P*	HR (95%CI)
Sex (male)	**0.039**	3.016(1.052-8.644)	0.119	2.394 (0.798-7.179)
Age (>65)	0.689	1.157(0.567-2.359)		
Treatment (surgery plus adjuvant therapy)	0.871	1.126(0.270-4.704)		
T-stage (T3-4)	0.524	1.474(0.448-4.849)		
MRI-reported LNM (+)	0.279	1.491(0.724-3.071)		
MRI-reported EMVI (+)	**0.012**	2.042(0.976-4.274)	**0.031**	1.099 (0.462-2.616)
CA199(>37)	0.570	1.414(0.431-4.643)		
CEA (>5)	0.240	1.539(0.752-3.149)		
Tumor length (>44mm)	0.139	1.753(0.837-3.671)		
Wall thickness (>13mm)	0.907	0.957(0.462-1.980)		
Radscore 3 (>-0.052)	0.483	1.295(0.629-2.668)		
Clinical-radiomic nomogram-based LNM (> 63)*	**0.004**	3.192(1.457-6.990)	**0.017**	2.232 (1.238-7.439)

HR, hazard ratio; CI, confidence interval; EMVI, extramural venous invasion; LNM, lymph node metastasis; radscore 3, combined intratumoral and peritumoral radiomic scores. Sex (Female), age (≤65), treatment (surgery only), T-stage (T1-2), MRI-reported LNM(-), EMVI (−), CA199(≤37), CEA (≤5), tumor length (≤44mm), wall thickness (≤13mm), radscore 3 (≤-0.052) and clinical-radiomic nomogram-based LNM (≤63) were as a reference in univariate and multivariate Cox analysis. The bold for P-value means there is a significant difference. *indicates the cutoff value of 63 for Clinial-radiomics nomogram score for differentiating LNM+ from LNM-.

## Discussion

In the current study, the radscore 3 outperformed the radscore 1 and radscore 2 for identifying LNM. After adding the radscore 3 model to the clinical model, our study revealed that the clinical-radiomics nomogram could significantly improve diagnostic performance compared to the clinical model in training cohort and internal validation cohort. However, the clinical-radiomic nomogram failed to outperform the clinical model in external validation cohort, but the difference was not significant. Moreover, prognostic model constructed by MRI-reported EMVI and clinical-radiomic nomogram-based LNM indicated good performance for predicting 3-year RFS.

The radscore 3 consisting of 10 radiomics features could predict LNM with acceptable performance in training cohort (AUC of 0.77) and internal validation cohort (AUC of 0.76). Of the 10 radiomics features, peritumoral features accounted for most of the features in radscore 3 (6/10, 60%). In this study, the two important positive coefficients of radiomics features included interquartile range and skewness extracted from peritumoral region. Interquartile range is the 25th and 75th percentile of the image array, respectively. The large interquartile range indicates the greater difference between the range of gray values in the region of interest, which implies the inhomogeneous intensity of tumor. Therefore, the two positive coefficient of radiomic features indicating the tumor heterogeneity suggested that the peritumoral region around the rectum is important in the formation of LNM. Our study found that radscore 3 model showed minor improvements in diagnostic efficacy compared with radscore 1 and radscore 2 model. Wavelet features are extracted from the images transformed by a wavelet filter. Consistent with a previous study (25), the selected radiomic signature in this study was mainly constructed by wavelet features (6/10, 60%). Another study also reported the effectiveness of wavelet features on T2WI in predicting lymph node status (26). Therefore, these results confirmed that wavelet features better reflected tumor heterogeneity. Some studies have reported that some clinical characteristics are related to LNM (27–29). Our study found that cT stage, MRI-reported EMVI and CEA were independent predictors for LNM, suggesting that patients with rectal cancer with LNM are more likely to have a high T stage, CEA level or EMVI+. Moreover, a model derived from these clinical characteristics had a slightly higher AUC than that of the nomogram in external cohort (0.76 vs. 0.72). This result could be attributed to the characteristics of the study population itself as greater than half of patients in external cohort have LNM (88/156, 56.4%).

Most previous studies based on radiomics have mainly focused on features from intratumoral regions in rectal cancer. Huang et al. showed that a CT-based radiomics nomogram can be used to facilitate the preoperative prediction of LNM with a concordance index of 0.736–0.778 (12). Meng et al. reported that incorporating a multiparametric radiomic signature and MRI-reported LN status had an AUC of 0.697 (29). Compared with these studies, the AUC of the nomogram in our study was slightly higher. This finding could be explained by the multiregional radiomics feature extraction in our study. Other previous studies reported that a multiparametric MRI-based radiomics nomogram for the tumor region alone showed a slightly improved diagnostic performance compared with that noted in our study (14, 25). However, the sample size in these studies was relatively small, and these retrospective studies lacked independent external validation. Moreover, multiparametric MRI-based radiomics, especially for incorporating DWI-based radiomics features that could be influenced by MRI systems or b-values, is not stable and typically exhibits different diagnostic performance. Total mesorectal excision was introduced to reduce the local recurrence because the probable microtumors around the cancer have been completely removed. Therefore, the importance of the perirectal tissue status may possess some crucial biological information, including potential predictive markers. Liu et al. demonstrated that clinical data combined with multiregional-based MRI radiomics can improve the diagnostic efficacy in predicting LNM (30). Jayaprakasam et al. reported that radiomics features of mesorectal fat can predict tumor response after neoadjuvant chemoradiation therapy (31). However, peritumoral regions in this study were defined the region along the mesorectal fascia and the outer edge of the tumor and rectal wall. Several previous studies in other tumors indicated that peritumoral regions were defined as the area immediately surrounding the tumor (18, 20, 32, 33). Some studies also reported metabolic changes in the peritumoral region, including increased uptake of FDG by the tissues adjacent to the tumor compared with distant tissues (34, 35). Therefore, we chose a 3-mm area around the tumor boundary as the peritumoral region according to previous studies (18, 20).

Regarding subgroup analyses, patients with LNM≥4 (stage N2) had at least stage III rectal cancer (36). Different treatments and outcomes are noted between stage I-II and III rectal cancer patients. Our results showed that the nomogram had moderate value for differentiating N2 stage from LNM-negative patients. ENE was associated with a poorer prognosis in colorectal cancer patients (37). The nomogram had good diagnostic performance (AUC, 0.837) for differentiating ENE-positive from LNM-negative patients. Our study showed that the nomogram had moderate diagnostic performance (AUC, 0.752) for differentiating LLN-positive from LNM-negative patients. At T1-T2 stage subgroup analysis, we found that the nomogram had good diagnostic performance for identifying LNM (AUC, 0.813). In T3 stage subgroup analysis, the nomogram had moderate diagnostic performance for identifying LNM (AUC, 0.739). Although the sample size in these subgroup analyses was small, our study provided preliminary evidence to confirm that the nomogram could potentially assess these subgroups. In addition, we further reported that the rate of recurrence in patients with LNM was increased compared with that noted in those without LNM (51.9% vs. 8.3%). We found the patients with low clinical-radiomic nomogram score had better 3-year RFS than those with high scores. MRI-reported EMVI has been confirmed to be strongly associated with distant recurrence (38). In this study, multivariate Cox analysis showed MRI-reported EMVI and clinical-radiomic nomogram-based LNM were shown to be adverse prognostic factors for 3-year RFS. Prognostic model constructed by these two indicators indicated good performance for predicting 3-year RFS. These results may indicate T-stage and N-stage are not enough for classifying the patient, while combination of more indicators, such as clinical-radiomic nomogram-based LNM and MRI-reported EMVI, is more sensible.

Our study has several limitations. First, the sample size was relatively small, especially in the subgroup analysis. It is still necessary to expand the sample size in further study. Second, although a dilation distance of 3 mm around the tumor was defined as the peritumoral region in this study, we did not compare the different dilation distances. Third, the data were obtained from two different centers with different scanning devices. However, ComBat harmonization was used to efficiently remove the scanner/protocol effect. Fourth, even though DWI is routinely included in rectal MRI protocols and offers several benefits in various applications, it also has multiple possible shortcomings. Manual drawing of ROIs onto the tumor for quantitative or qualitative assessment may result in interobserver variation. Furthermore, image distortion due to artifacts is common on DWI, particularly around air tissue interfaces. These shortcomings may interfere with radiologists in drawing tumor ROI. Finally, our findings are applicable to resectable rectal cancer, whereas patients who had a contraindication for surgery were excluded. Therefore, a selection bias might exist.

In conclusion, our study confirmed clinical-radiomics nomogram exhibits good clinical potential for predicting preoperative LNM. Prognostic model constructed by MRI-reported EMVI and clinical-radiomic nomogram-based LNM indicated good performance for predicting 3-year RFS. These results can assist predicting preoperative LNM and identifying high-risk patients with rectal cancer for assessing 3-year RFS.

## Data availability statement

The raw data supporting the conclusions of this article will be made available by the authors, without undue reservation.

## Ethics statement

This prospective study was approved by the institutional review board of Sichuan Provincial People’s Hospital. The patients/participants provided their written informed consent to participate in this study.

## Author contributions

ZL-L and TL directed the project and revised the paper. HaL and X-LC conceptualized and designed the study, analyzed the data, and wrote the paper. HuL wrote section of the manuscript. HL analyzed the data. All authors contributed to the article and approved the submitted version.
