# Supplementary material for: MRI-based multiregional radiomics for predicting lymph nodes status and prognosis in patients with resectable rectal cancer
Source: Front Oncol. 2023 Jan 4;12:1087882. doi: 10.3389/fonc.2022.1087882 (PMC9846353; doi:10.3389/fonc.2022.1087882)
Supplement: Supplementary file 1 [file Image_1.pdf]

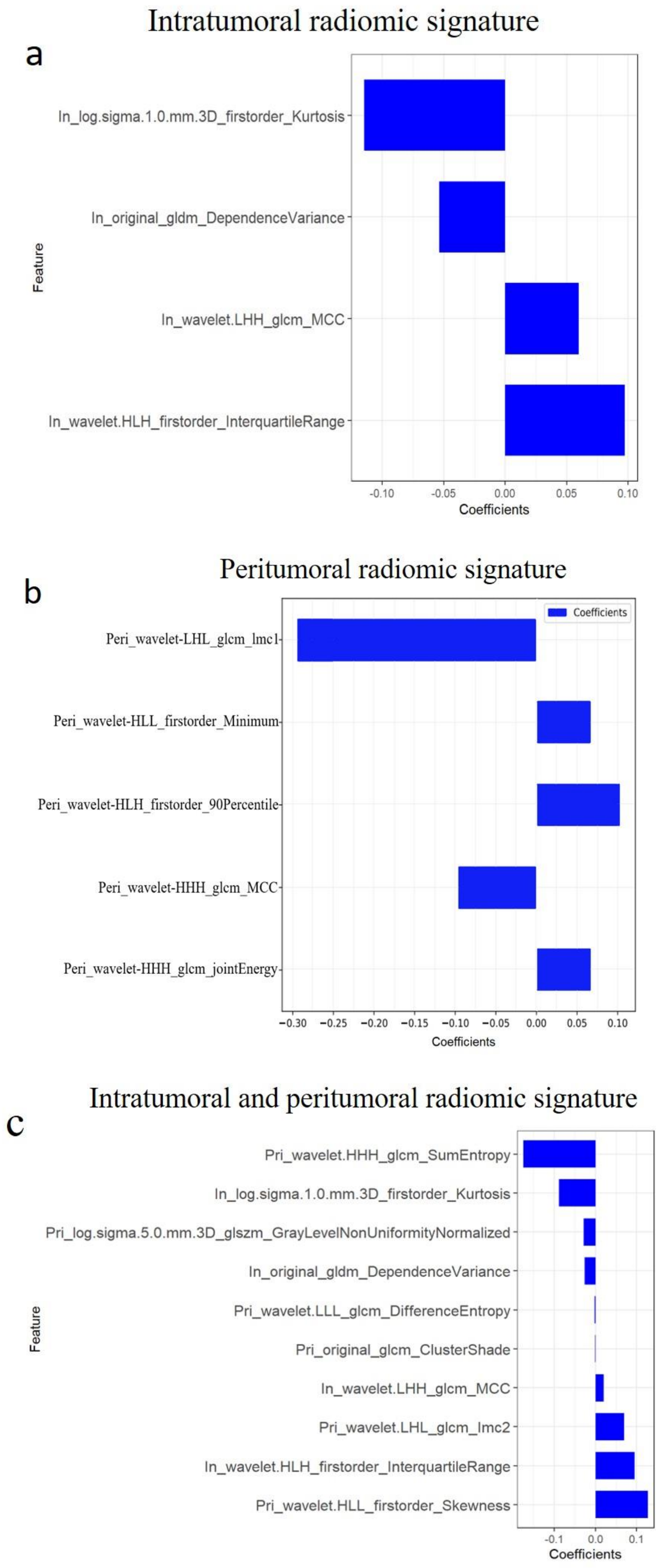

Supplementary Figure 1 The selected radiomic features weighted by their respective coefficients.
