# Supplementary material for: MRI-based multiregional radiomics for predicting lymph nodes status and prognosis in patients with resectable rectal cancer
Source: Front Oncol. 2023 Jan 4;12:1087882. doi: 10.3389/fonc.2022.1087882 (PMC9846353; doi:10.3389/fonc.2022.1087882)
Supplement: Supplementary file 2 [file Table_1.docx]

| **Supplementary Table 1** The correlation between MRI-reported LNM and pathological-reported LNM | | | |
| --- | --- | --- | --- |
| MRI-reported LNM | Pathological-reported  LNM+ (n=181) | Pathological-reported LNM- (n=165) | *P-*value |
|  |  |  | <0.001 |
| Negative | 68(37.6) | 103(62.4) |  |
| Positive | 113(62.4) | 62(37.6) |  |
| Note: LNM, lymph node metastasis. | | | |
